# Comparative transcriptome analysis on drought stress-induced floral formation of *Curcuma kwangsiensis*

**DOI:** 10.1080/15592324.2022.2114642

**Published:** 2022-10-03

**Authors:** Xin Feng, Liying Zhou, Aiwu Sheng, Ling Lin, Huicheng Liu

**Affiliations:** Department of Horticulture and Landscape Architecture, Zhongkai University of Agriculture and Engineering, Guangzhou, China

**Keywords:** Drought stress, flower induction, *Curcuma kwangsiensis*, drought escape, transcriptome

## Abstract

The rhizomes and tubers of *Curcuma kwangsiensis* have extensive medicinal value in China. However, the inflorescences of *C. kwangsiensis* are rarely known in horticulture, because of its low field flowering rate. In order to improve the flowering rate of *C. kwangsiensis*, we conducted drought stress treatment on the rhizome of *C. kwangsiensis*. The flowering rate of rhizome was the highest after 4d of drought stress treatment, and the buds on the rhizome could be obviously swell on the 4th day of rehydration culture. In order to identify the genes regulating the flowering time of *Curcuma kwangsiensis*, comparative transcriptome analysis was performed on the buds on rhizomes before drought stress treatment, 4 d after drought stress treatment and 4 d after rehydration culture. During this process, a total of 20 DEGs controlling flowering time and 23 DEGs involved in ABA synthesis and signal transduction were identified, which might regulate the flowering of *C. kwangsiensis* under drought stress. Some floral integration factors, such as *SOC1* and *FTIP*, were up-regulated under drought stress for 4 d, indicating that *C. kwangsiensis* had flowering trend under drought stress. The results of the present study will provide theoretical support for the application of *Curcuma kwangsiensis* in gardening.

## 1. Introduction

The genus *Curcuma* (family *Zingiberaceae*) comprises more than 80 species of rhizomatous herbs and widely spread in the tropics of Asia to Africa and Australia.^1^ As a source plant of traditional medicine, *Curcuma kwangsiensis* is widely cultivated in southwestern China. According to the ornamental value by its green slender foliage and colorful cylindrical inflorescences, it is also commonly used as bedding plant, pot or hydroponic flower and cut flower with over 3–4 weeks of post-harvest life.^2^ The natural flowering season of *C. kwangsiensis* concentrated in May – June and August – September, but the flowering rate was only 5% under field conditions when it cultivated as medicinal plants, which seriously limits the commercial application of *C. kwangsiensis* inflorescence. Previous studies showed that sufficient low-temperature dormancy time and cultured at 30°C can greatly improve the flowering rate of rhizome of *C. kwangsiensis* var *nanlingensis*.^3^ Compared with cultivated at 14-h photoperiod, the flowering rate of *C. alismatifolia* Gagnep. significantly reduced under the short-day environment of 6- and 10-h photoperiod, which indicated that photoperiod was also one of the factors affecting the flowering rate of *Curcuma* plants.^4,5^ Despite little is known about the mechanism of these induced flowering of *Curcuma* by changing environmental conditions, existing studies have shown that the application of appropriate cultivation techniques to improve the flowering rate of *Curcuma* seems to be a feasible strategy.

Flowering is regulated autonomously or by environmental factors.^6^ In tropical and subtropical regions, drought stress has obvious induction effect on flower bud differentiation in some evergreen fruit trees.^7^ This early flowering phenomenon due to water deficit is referred to as drought escape (DE).^8^ Horticulturalists are able to promote flowering by limiting irrigation during cultivation,^9^ which has been successfully applied to *Pharbitis*,^10^ coffee,^11^ lemon^12^ and other plants in recent years. Early flowering due to lack of water in the environment not only shortens the growth period of plants, but also saves irrigation costs. More importantly, this method of regulating flowering is non-chemical and absolutely safe.

Studies on the model plant *Arabidopsis thaliana* show that drought escape is mediated by abscisic acid, because the *aba1* mutant of *A. thaliana* shows a decrease in the expression of flowering integration factor *FT* under drought conditions.^13–15^ In addition, drought stress is often accompanied by other regulatory pathways in plant flowering induction. In *Arabidopsis*, water deficit promoted flowering under long-day conditions and delayed flowering under short-day conditions, suggesting that drought-mediated regulation was related to photoperiodic flowering pathways.^16^ Further studies have shown that drought can up-regulate the transcription level of photoperiod-sensitive gene *GI*, and early flowering caused by drought escape cannot occur in *gi* mutant, indicating that *GI* plays an important role in drought escape.^17^

Although the evidence that stress is a flower-inducing factor is accumulating,^6^ it is still unclear whether drought stress affects the flowering of *C. kwangsiensis*. Previously used low temperature and long-day conditions to induce flower formation in *Curcuma*. Our aim was to promote the flowering of *C. kwangsiensis* by drought stress based on these studies. RNA-Seq technology was used to conduct a comparative transcriptome analysis of buds, focusing on the expression patterns of flowering related genes in several critical periods of drought stress. In addition, we also analyzed the changes of ABA in several key periods to determine whether drought escape had occurred in buds from physiological and biochemical perspectives. This study will provide theoretical guidance for understanding the flowering regulation mechanism under drought stress and promoting the cultivation and horticultural application of *C. kwangsiensis*.

## 2. Materials and methods

### 2.1. Plant materials

Underground rhizomes of *C. kwangsiensis* after winter withering were used as experimental material. The rhizomes were first harvested in Nanning, Guangxi Zhuang Autonomous Region, and then stored at (13 ± 2°C, 65 ± 5% RH) river sand (sterilized by high-pressure steam, and maintaining water content of 10–15%) until use. After 40d of cold storage, storage temperature adjusted to 30°C for 20d to break rhizome dormancy and induce the germination of the rhizome buds.

### 2.2. Drought stress treatments

When the buds on the rhizome sprout to 3 cm, rhizomes were moved from the river sand to the glass culture dish waiting for treatment. The rhizomes were divided into three groups, one group of rhizomes was directly hydroponically cultured in glass culture dish with distilled water, as a control treatment and denoted as DT0. Other rhizomes placed in petri dishes to remain the state of water-free for drought stress. When the rhizomes experienced continuous drought stress for 4 d and 8 d, they began to rehydration cultured in distilled water, which were denoted as DT4 and DT8, respectively. In hydroponic culture, the height of distilled water was less than 2/3 of the rhizome height, and water was changed every other day. The culture condition was 30°C, and maintaing full illumination at all times with light intensity 3500 Lx. In several critical periods of drought stress and rehydration, sampled buds were sequenced. The three sampling periods were before the drought stress treatment (pro-drought stress treatment, PDT), drought stress treatment for 4 d (DT), and rehydration culture for 4 d after 4 d drought stress treatment (rehydration treatment, RT). Four plant buds were pooled as one sample and immediately flash-frozen in liquid nitrogen and stored at −80°C for subsequent experiments. Three biological replicates were collected for each sampling time.

### 2.3. RNA isolation and high-through sequencing

Total RNA was extracted using MiniBEST Plant RNA Extraction Kit (TaKaRa, Japan) according to the manufacturer’s protocol. RNA quality was assessed using a 1% agarose gel and RNA Nano 6000 Assay Kit on an Agilent Bioanalyzer 2100 system (Agilent Technologies, CA, USA). Sequencing libraries were generated using the Library Prep Kit, following the manufacturer’s instructions (#E7530, New England Biolabs, MA, USA). RNA-seq was then performed using the Illumina HiSeq TM 4000 platform, by Gene Denovo Biotechnology Co. (Guangzhou, China).

### 2.4. Gene expression levels and differentially expressed genes analyses

After filtering the raw reads, transcriptome de novo assembly was conducted with the short-read assembly program Trinity (v.2.8.4, https://github.com/trinityrnaseq/trinityrnaseq).^18 T^he resulting Trinity sequences were called unigenes. Unigene annotation was performed using various bioinformatics databases, including the non-redundant protein database (Nr), gene ontology database (GO), cluster of orthologous groups of proteins database (COG), Kyoto encyclopedia of genes and genomes database (KEGG), and Swiss-Prot protein database (Swiss-Prot). Transcript abundance was estimated using the fragments per kilobase of transcript per million mapped reads (FPKM) method. DESeq was used to identify differentially expressed genes (DEGs) between the samples.^19^ The DEGs between two samples were determined based on a log_2_-fold change (FC) of at least ± 1 and a false discovery rate <0.01.

### 2.5. Validation of RNA-Seq data by quantitative real-time PCR

First-strand cDNA synthesis was performed using the HiScript II 1st Strand cDNA Synthesis reagent with a gDNA wiper kit (Vazyme Biotech, Nanjing, China) according to the manufacturer’s instructions. Quantitative real-time PCR (qRT-PCR) was carried out using 2 × Real Star SYBR Mixture with ROX II (Genestar, Shanghai, China) on a Real-Time PCR System (CFX384, Bio-Rad) with the following program: one cycle of 95°C (2 min), 40 cycles of 95°C (10 s) and 60°C (30 s). Relative mRNA levels were calculated using the 2^−ΔΔCt^ method.^20^

### 2.6. Determination of ABA content

Endogenous ABA content of buds at three critical stages of pre-and-post-drought stress was determined, using the ELISA Kits purchased from Shanghai Enzyme-linked Biotechnology Co., Ltd. For the extract of ABA, six individual buds were sampled at each stage as replicates, and 0.3 g fresh buds were used for each replicate per treatment. The SPSS (Statistical Product and Service Solutions, Chicago, USA, 2008) was used to determine physiological differences among means of treatment. These differences were assessed using Fisher’s protected least significant difference test (LSD) at *p *≤ .05.

## 3. Results

### 3.1. Effects of different drought stress on flowering

**Figure 1.**
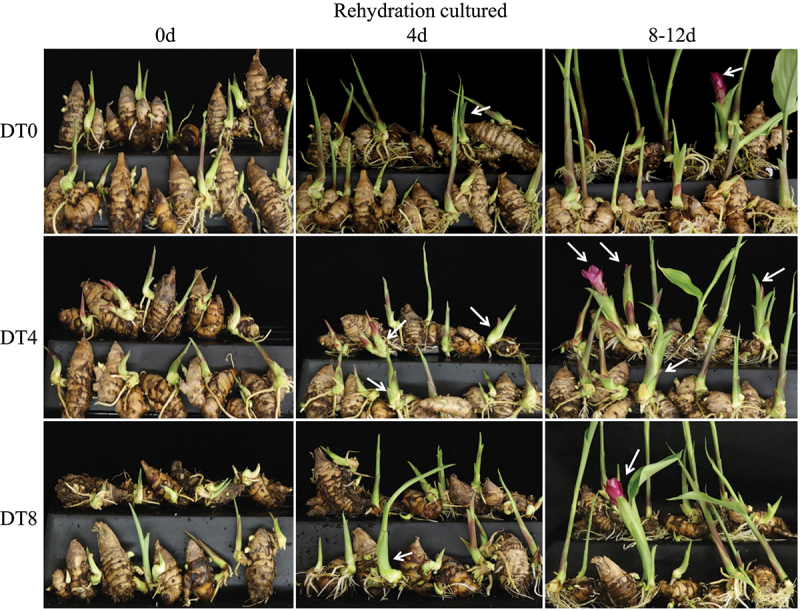
Rehydration culture of *Curcuma kwangsiensis* under different drought stress. DT0, Direct rehydration culture without drought stress. DT4, Rehydration culture after 4 d of drought stress. DT8, Rehydration culture after 8 d of drought stress. White arrows indicate that the base enlargement of the bud or the bud develops into inflorescence.

After 0d, 4d and 8d drought stress treatment (denoted as DT0, DT4, DT8, respectively), the rhizomes of *C. kwangsiensis* were cultured in water environment, and 3 groups showed different flowering rate, initial flowering time and the time interval between inflorescence and leaf expansion (Table 1, Figure 1).

**Table 1.** Effects of Different Drought Stress on Flowering.

	DT0	DT4	DT8
Final flowering rate	23.3%	43.3%	26.67%
Initial flower time after rehydration culture	12d	8d	10d
Time interval between inflorescence and leaf expansion	17d	20d	18d

DT4-treated showed the highest flowering rate (43.3%), which was 20% higher than DT0-treated (Table 1). The flowering rate of DT8-treated changed little compared with DT0. DT4-treated rhizome began to appear inflorescence on the 8th day after rehydrated culture. However, the inflorescence appear time of the DT0 and DT8 treatment groups after rehydrated culture was 12th and 10th days, respectively (Table 1). The interval time from inflorescence to leaf expansion of the 3 treatment groups was counted, and it was found that DT4-treated plant had the longest interval time. At the same time, the interval time of DT0 and DT8 treatment groups was 17d and 18d, respectively (Table 1). In summary, in the process of rehydration culture of rhizomes treated with drought stress for 4 d, the final flowering rate of buds was the highest, the flower buds appeared the earliest, and the interval between inflorescences and leaves was the longest. From these results, it can be known that 4d drought stress treatment is conducive to the flower formation of *C. kwangsiensis*.

In addition, by observing the changes of buds on rhizomes during rehydration culture, it was found that the buds would swell at the base on the 4th day of rehydration culture (Figure 1). This phenomenon was observed in bud materials of 3 treatment groups. Based on this phenomenon, buds may be undergoing important flower bud differentiation process at this time.

### 3.2. Sequence analysis and assembly

To generate a broad survey of genes involved in flowering regulation induced by drought stress and rehydration, nine mRNA libraries were constructed from buds of *C. kwangsiensis* at three different time points: before the drought stress treatment (pro-drought stress treatment, PDT), drought stress treatment for 4 d (DT), and rehydration culture for 4 d after 4 d drought stress treatment (rehydration treatment, RT).

After removing the reads with adapters, low-quality and high content of unknown base (N), the basic information (including clean reads, Q20, Q30, and GC% of each sample) was recorded based on transcriptomic analysis (Table 2).

**Table 2.** Sample sequencing data evaluation statistical table.

Samples	Reads number	Bases (bp)	Q30 (%)	GC content (%)
PDT-1	51929720	7752131128	93.70	46.17
PDT-2	48856946	7296328814	93.18	46.24
PDT-3	44424716	6620436581	94.00	46.21
DT-1	47099064	7030152196	94.06	45.38
DT-2	45885324	6848585822	93.92	45.26
DT-3	46381470	6932251456	94.10	45.34
RT-1	48415790	7232774239	93.84	46.00
RT-2	51646092	7715422027	94.05	45.98
RT-3	53937382	8058448629	93.67	46.31

A total of 65.16 Gb of clean data were generated from 9 libraries, each library approximately generated 6.62 Gb of nucleotide data with a Q30 (percentage of sequences with sequencing error rate lower than 0.1%) above 93.18%.

Then a total of 119620 unigenes were assembled by Trinity with a average length of 961 bp (N50 1807bp) (Table 3). Unigene length was in the range of 200–2000 nt, and the total length was 115023115 nt (Table 3; Figure S1). Distribution of the unigene lengths are shown in Figure S1.

**Table 3.** Summary statistics of assembled gene sequences.

Term	≥200 bp	≥500 bp	≥1000 bp	Total number	Total length (nt)	Mean length	N50
Unigenes	61384	26359	31517	119620	115023115	961	1807

### 3.3. Sequence functional annotation and classification

The 119620 unigene sequences obtained by sequencing were aligned against the protein database NR, KEGG, SwissProt, KOG/COG. The number of annotated unigene in the four databases was 51311, 44580, 33102, and 27386, respectively (Figure S2). The total number of annotated sequences was 52988, accounting for 44.29% of the all sequencing unigenes. In terms of distributed species, the homologous genes matched with the unique sequences of *C. kwangsiensis* were mainly concentrated in *Musa acuminata* (39.47%), followed by *Elaeis guineenis* (4.13%), *Phoenix dactylifera* (3.37%) (Figure S3).

**Figure 2.**
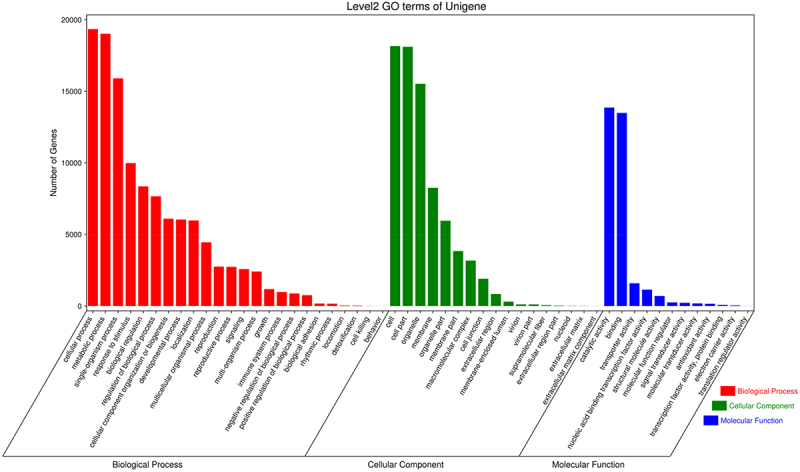
GO annotation statistics of *Curcuma kwangsiensis* unigenes.

We further classified the functions of all unigenes by GO assignment, a total of 23737 genes were categorized into three main GO categories, including 17 in cellular components, 12 in molecular functions, and 24 in biological processes (Figure 2). All the unigenes of *C. kwangsiensis* were analyzed in KEGG pathway database. Finally, 11159 unigenges were assigned to 5 main categories including 138 KEGG pathways. Metabolic pathways (ko01100, 42.94%), biosynthesis of secondary metabolism (ko01110, 22.89%) and ribosome (ko03010, 7.93%) were the main enriched pathways (Table S1).

### 3.4. Transcriptome in response to drought stress and rehydration

To investigate the differences among nine libraries, DESeq was used to identify differentially expressed genes (DEGs). Differences in gene expression were evaluted based on a false discovery rate <0.01 and a log_2_-fold change (FC) of at least ± 1. A total of 11807 DEGs were identified at the 3 different time point, i.e., 6123, 5458, and 6952 DEGs from pairwise comparisons of PDT-vs-DT, PDT-vs-RT, DT-vs-RT, respectively. Genes differentially expressed between PDT and DT, RT were screened out and there were many genes showing signifcantly different expression levels.

**Figure 3.**
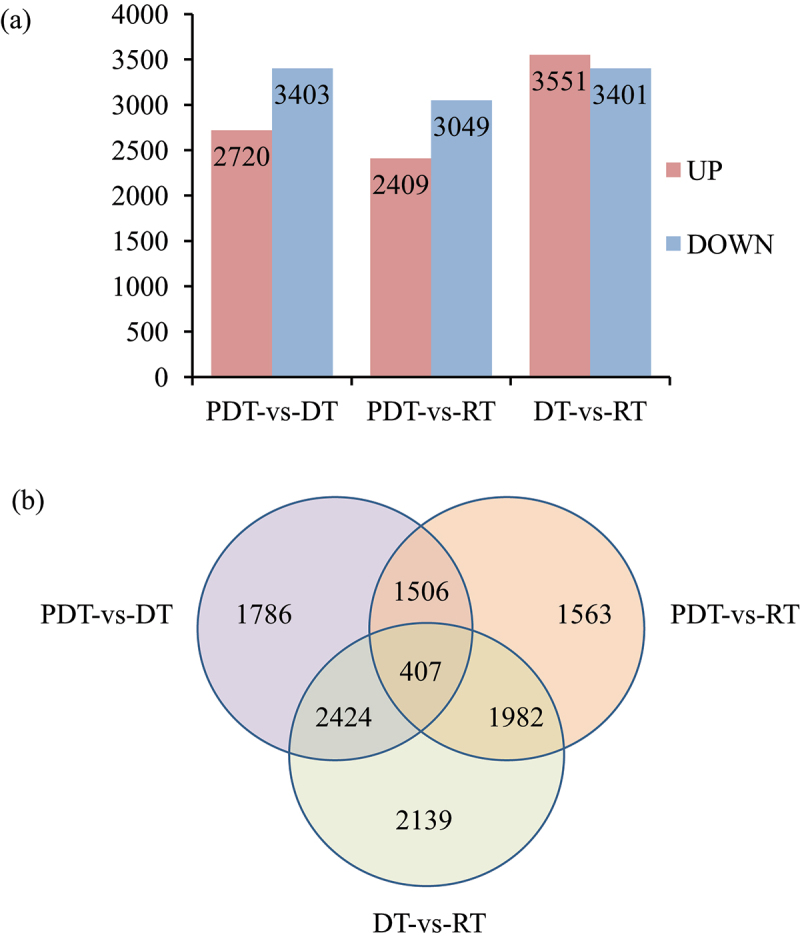
Differentially expressed genes (DEGs) identified under drought stress and rhydration (a) Statistic of differentially expressed genes (b) Venn diagram showing the number of DEGs in the three paired comparison.

The highest total number of the DEGs was identified in group of DT-vs-RT (Figure 3a). Among the three-component comparison, 407 differential genes were common (Figure 3b).

### 3.5. Identification of DEGs involved in flowering time

In the study of the flowering mechanism of plant, most of the key genes are involved in photoperiodic pathway, autonomous pathway, vernalization pathway, gibberellin pathway, age pathway, and temperature pathway. We identified 20 DEGs that may exercise flowering regulation function, mainly involving photoperiod pathway, vernalization pathway and autonomous pathway (Table 4). Most of the DEGs are involved in photoperiod flowering pathway, such as *CONSTANS-LIKE* (*COL), GIGANTEA* (*GI), SUPPRESSOR OF OVEREXPRESSION OF CO 1* (*SOC1*) and *FLOWERING LOCUS D* (*FD)*, indicating that drought stress and rehydration may regulate the flowering of *C. kwangsiensis* mainly by photoperiod pathway.

**Table 4.** Statistical results of DEGs invovled in flowering regulation.

Gene ID	Symbol	FPKM			Log2 fold change		
		PDT	DT	RT	DT/PDT	RT/PDT	RT/DT
Ck0047859	*COL2*	12.9233	1.25	3.2533	−3.36998	−1.98999	1.379991
Ck0006303	*COL5*	11.5733	2.3267	3.12	−2.31447	−1.89119	0.423281
Ck0082389	*COL9*	16.2867	7.6533	10.6167	−1.08953	−0.61736	0.472171
Ck0005261	*COL10*	10.2133	6.8567	14.2367	−0.57487	0.479158	1.054032
Ck0112117	*COL14*	1.48	1.1333	3.7867	−0.38502493	1.355331	1.740356
Ck0024125	*COL16*	9.18	1.6467	4.95	−2.47895	−0.89107	1.58788
Ck0042063	*COL16*	20.29	2.0633	3.7867	−3.29772	−2.42177	0.875952
Ck0082850	*COL16*	8.7867	0.7133	4.45	−3.62267	−0.98151	2.641157
Ck0065587	*GI*	2.3467	4.8567	3.1167	1.0488	0.4067	0.6421
Ck0118854	*SOC1*	1.7733	3.7833	3.3133	1.093194	0.90182	−0.19137
Ck0010155	*SOC1*	5.6033	15.7267	15.11	1.488856	1.431146	−0.05771
Ck0027021	*SOC1*	1.3467	7.6	5.6367	2.496607	2.065449	−0.43116
Ck0045971	*SOC1*	11.9567	36.26	19.3867	1.600564	0.69725	−0.90331
Ck0005882	*FD*	4.48	16.3033	21.4733	1.863596	2.260976	0.397379
Ck0115713	*UFC*	8.24	3.6033	2.61	−1.19331	−1.65859	−0.46528
Ck0006478	*LHY*	22.4267	7.7333	2.1533	−1.53605	−3.38057	−1.84452
Ck0006587	*LHY*	61.94	21.0533	7.02	−1.55682	−3.14133	−1.58451
Ck0009620	*ELF3*	0.8233	0.11	0.7133	−2.90397	−0.2069	2.697073
Ck0019762	*FCA*	2.3967	6.3033	5.7933	1.395086	1.273364	−0.12172
Ck0117708	*FTIP*	16.6133	45.4467	22.0067	1.451833	0.405599	−1.04623

A total of 8 *COL* genes were differentially expressed. Except for *COL10* and *COL14* expression increased in RT, others (*COL2, COL5, COL9, COL16*) showed the highest expression level in PDT. In addition, the increased expression of *GI* and *SOC1* involved in photoperiod pathway were detected in DT and RT.

In vernalization pathway, the encoding gene of *UPSTREAM OF FLC-like* protein (*UFC*) showed the highest expression level in PDT, and decreased in DT and RT.

In the plant rhythm pathway, we detected an decreased in the expression of *EARLY FLOWERING 3* (*ELF3*) and *LATE ELONGATED HYPOCOTYL* (*LHY*) in DT and RT. Floral integration factors, *FT* interacting protein (*FTIP*), expressed higher in DT or RT than PDT.

### 3.6. Identification of DEGs involved in ABA signaling

**Figure 4.**
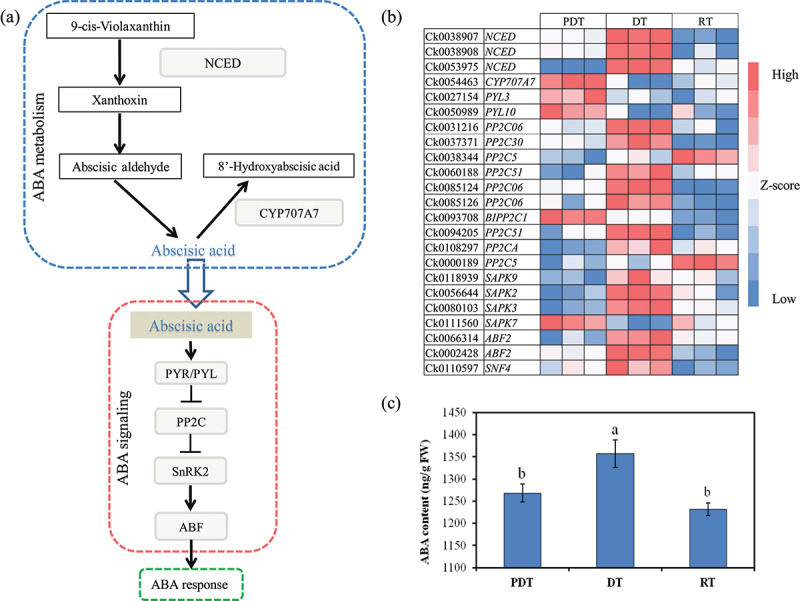
Differential expression of genes involved in ABA synthesis and signal transduction in C. kwangsiensis buds as reconstructed from transcriptomic evidence. (a) Model of the differential expression of genes involved in ABA synthesis and signal transduction. (b) Heat map of DEGs assigned to ABA synthesis and signal transduction. (c) ABA content of C. kwangsiensis buds in different stage.

ABA plays an important role in response to drought stress and flowering regulation. In the ABA metabolic pathway, *putative 9-cis-epoxycarotenoid dioxygenase 3* (*NCED*) is the key rate-limiting enzyme in the biosynthesis of ABA, while *abscisic acid 8’-hydroxylase* (*CYP707A7*) controls the degradation of ABA (Figure 4a). In the presence of ABA, the ABA signaling complex consisting of ABA receptor protein *PYL*, protein *phosphatase 2CA* (*PP2CA*) and *SNF1-RLATED PROTEIN KINASE 2* (*SnRK2*) was activated (Figure 4a). Based on the FPKM value, we identified 23 DEGs involved in ABA metabolism and signal transduction, among which 4 were involved in ABA metabolism, including *NCED* and *CYP707A7*, and the other 19 were related to ABA signal transduction, including *PP2C, PYL* and *SnRk2* constituting ABA signal complexes (Table S2, Figure 4b). Differential expression of genes in ABA metabolism and signal transduction pathway indicated that drought stress and rehydration caused the changes in endogenous ABA level of *C. kwangsinesis* buds and triggered ABA signal transduction is activated.

In ABA metabolism pathways, three encoding genes of *NCED* were up-regulated at DT, and one encoding gene of *CYP707A7* was down-regulated at DT and RT (Figure 4b), indicating that the endogenous ABA content in the buds of *C. kwangsiensis* might maintain at a high level under drought stress or rehydration conditions. This view is also supported by the determination of endogenous ABA content in buds at 3 time points (Figure 4c).

Among the 19 DEGs involved in ABA signal transduction, the encoding genes of each ABA signal response receptor can be divided into three categories according to their expression levels: (1) DEGs with the highest expression level in PDT such as *PYL3, PYL10, BIPP2C1* and *SAPK7*; (2) DEGs with the highest expression level in DT, including *PP2C06, PP2C30, PP2C51, PP2CA, SAPK2, SAPK3, SAPK9, ABF2* and *SNF4*; (3) DEGs with the highest expression level in RT, only *PP2C5*. Obviously, most genes encoding ABA signaling receptors are up-regulated at DT, while some negatively regulated genes are down-regulated at DT (Figure 4b).

### 3.7. Gene expression changes analysis by qRT-PCR

**Figure 5.**
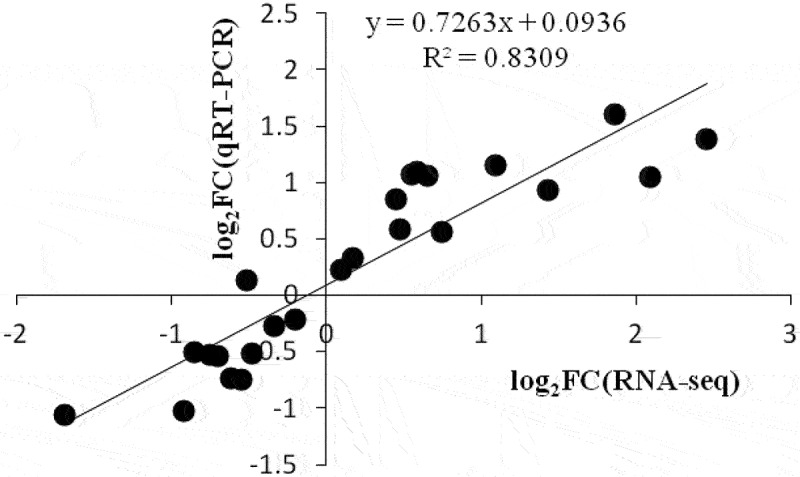
Correlation analysis between RNA-seq and qRT-PCR data. Eight DEGs from the RNA-seq assay were used for qRT-PCR assay. The log_2_FC obtained by RNA-seq (X-axis) was plotted against log_2_FC by qRT-PCR (Y-axis).

To validate the accuracy and reliability of the RNA-Seq data, we monitored the expression of 8 DEGs selected randomly by qRT-PCR evaluation (Table S3). Correlation analysis of the gene expression ratios showed a good correlation (R^2^ = 0.8309) between RNA-Seq and qRT-PCR, indicating the high reliability of the RNA-Seq data obtained in our study (Figure 5).

## 4. Discussion

Flowering is induced by endogenous signals and environmental factors, including photoperiod, vernalization, temperature, phytohormones, and etc. These signals and factors cross-linked with each other for a complex regulatory network, and initiating plant flowering.^21^ In the case of *curcuma* genus, low natural flowering rate limits the horticultural application of rhizome. With the aim of improving flowering rate, previous studies have confirmed that low-temperature storage of rhizomes, and cultivation under high temperature and long daylight conditions can be promote flowers of *curcuma* genus.^3,22–24^ Our study first showed the effect of moderate drought stress on flower induction of *C. kwangsiensis*. Compared with cultivated under sufficient water conditions, the buds on the rhizomes after 4 d drought stress had higher flowering rate and earlier flowering period (Table 1), indicating that moderate drought stress promotes bud transfer from vegetative to reproductive growth. This result is also supported by previous studies that plants tend to accelerate flowering and produce seeds in response to drought stress.^6,25,26^ However, buds finally flowered during rehydration of rhizomes, and we observed that buds would swell on the 4th day of rehydration (Figure 1). The flowering rate of *C. kwangsiensis* could not be improved effectively by continuous drought stress or water culture. Therefore, we believe that drought exercise resulted in a series of physiological changes and molecular events conducive to flower bud differentiation, and the differentiation completed finally under sufficient water condition. To further understand the complex molecular events, the changes in transcriptomes in buds under drought and rehydration treatments were studied using RNA-Seq technology.

Following the reason that flowering time has been shown to be directly related to grain yield, plant resources utilization, and ornamental value, many studies have explored flowering time in various plants.^27–30^ Transcriptome analysis is an effective approach to study and identify the genes participating in the flowering pathway.^31^ By using comparative transcriptome analysis, differentially expressed genes associated with flowering under drought environment in Chinese Tallow Tree,^32^ *Cassava*^33^ and *Litchi*^34^ were identified. Based on these studies, we were able to analyze expression of homologs of genes previously reported to be related to flowering time in other plants. In our study, DEGs produced by drought stress and rehydration are mainly involved in photoperiod pathway and vernalization pathway. It is noteworthy that, compared with PDT, the expression patterns of these flowering-related genes in DT and RT are the same. Genetic and molecular analyses in recent years have made it clear that the genetically definition pathways regulating flowering are not strictly separated.^21,35^ Instead there is increasing evidence for the floral integration factor integrates inputs from different flowering cascades and transmits the results to floral meristem characteristic genes at stem tips.^36^ Obviously, the floral integration factor (*SOC1, FTIP, FD*) of *C. kwangsiensis* were up-regulated under drought stress, indicating the effect of drought stress on the flowering of *C. kwangsiensis* from the transcriptional level. However, it is also important to understand how drought stress activates these floral integration factors.

If placed under unsuitable growth conditions, plants have a tendency to flower.^6^ This mechanism of avoiding adverse drought stress by shortening its life cycle is referred to as drought escape, which is also the reason why some plants show the early-flowering phenotype under drought conditions.^16–37^ Studies on *A. thaliana* showed that drought escape was mediated by ABA, which was related to photoperiodic flowering pathway.^38–40^ The expression of *FT* and *TSF* in leaves was reported to be induced by ABA via *GIGANTEA* (*GI*) in the drought escape response.^13,14^ As a key hormone in drought escape response, the accumulation of ABA in plants and the expression levels of related genes are correspondingly changed under drought stress.^17,41,42^ *NCED* plays a major role in ABA biosynthesis.^43,44^ The expression level of *NCED*, an encoding gene of key rate-limiting enzyme involved in ABA biosynthesis, increased under drought stress (Figure 4b), indicating that ABA content was induced by environmental drought stress. *CYP707A7* participated in the oxidative degradation of abscisic acid.^45,46^ Studies have found that *CYP707A7* is widely expressed in leaves, flowers and other tissues before flowering in Japanese rice, while it is low expressed in flower buds.^47^ The expression of *CYP707A7* in buds decreased after drought stress (Figure 4b), indicating that ABA metabolism was weakened. The expression of *NCED* and *CYP707A7* in buds of *C. kwangsiensis* showed that drought stress increased ABA content, triggering drought escape reaction and inducing buds to flower. According to Riboni, ABA stimulates *GI* and *CO* signaling to boost *FT* activation.^14^ Under drought stress, 1 unigene annotated as *GI* was highly expressed in buds of *C. kwangsiensis*, while eight unigenes annotated as *COL* were down-regulated (Table 4). This asynchronous phenomenon of *GI* and *COL* expression was also reported in earlier study.^39^ It was speculated that *GI* affected other photosensitive proteins to promote drought escape reaction, or ABA promoted the direct effect of *GI* on *FT* promoter in a manner independent of *CO*.^17^ In addition, studies have reported that *COL10* and *COL16* inhibit rice flowering by effecting on *Ghd7*,^48,49^ which is similar to the results observed in this study. In the present study, most ABA signaling genes such as *PP2Cs, SAPKs* and *ABFs* showed higher transcription levels in the buds of *C. kwangsiensis* under drought stress (Figure 4b). Thus, ABA signaling has a potential regulatory role in *C. kwangsiensis* flowering time, but it is unclear whether this positive effect on the expression of genes regulating flowering time can be directly or indirectly attributed to ABA.^31^

In summary, drought stress increased the probability of bud development to flower. By analyzing the buds during drought stress, it can be found that the flowering time-regulated genes are actively expressed at this time, and the ABA content is increased. Most genes related to ABA signal transduction and *GI*, a key gene in photoperiodic flowering pathway,^50^ were also activated and expressed, indicating the existence of drought escape response.

## 5. Conclusions

To our knowledge, this report is the first to provide comprehensive transcriptome analysis data related to flower bud differentiation of *C. kwangsiensis* during drought stress, and also the first to reveal the effect of drought stress on flower formation of *C. kwangsiensis*. Twenty candidate DEGs were identified that might play important roles in flowering time control. The differential expression of genes involved in ABA signaling pathway and flowering regulatory genes under drought stress increased our understanding of molecular events occurring in the process of drought escape. At the same time, our research provided theoretical support for artificially regulating the flowering of *C. kwangsiensis* in horticulture.

## Supplementary Material

Supplemental Material

## Data Availability

The raw sequencing data from this study have been deposited in the Genome Sequence Archive in BIG Data Center (http://bigd.big.ac.cn/), Beijing Institute of Genomics (BIG), Chineses Academy of Sciences, under the accession number: CRA005642.
