# Supplementary material for: Comparative transcriptome analysis on drought stress-induced floral formation of Curcuma kwangsiensis
Source: Plant Signal Behav. 2022 Oct 3;17(1):2114642. doi: 10.1080/15592324.2022.2114642 (PMC9542783; doi:10.1080/15592324.2022.2114642)
Supplement: Supplemental Material [file KPSB_A_2114642_SM7473.doc]

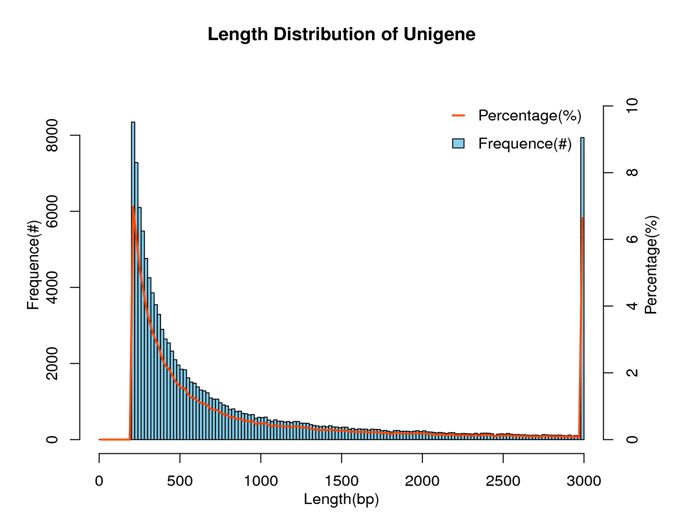
**Figure S1. Unigenes length statistics**


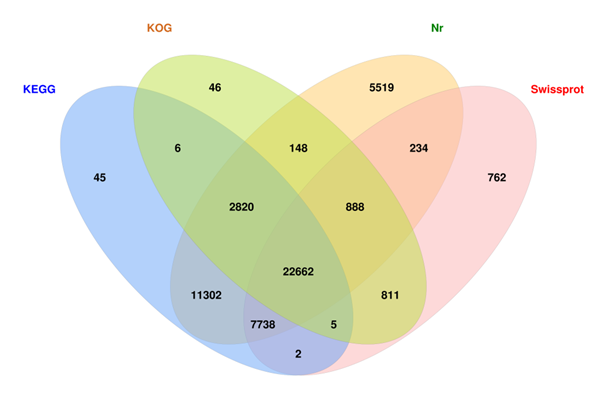


**Figure S2. Numbers of annotated unigenes by using different database**


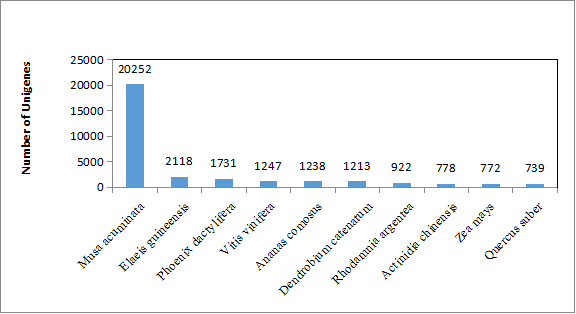


**Figure S3. The TOP 10 species matched Unigenes in NR database**

**Table S1 The TOP 10 pathway annotated Unigenes in KEGG database**

| Pathway | Number | Pathway ID |
| --- | --- | --- |
| Metabolic pathways | 4792 | ko01100 |
| Biosynthesis of secondary metabolites | 2554 | ko01110 |
| Ribosome | 885 | ko03010 |
| Carbon metabolism | 777 | ko01200 |
| Plant-pathogen interaction | 688 | ko04626 |
| Biosynthesis of amino acids | 657 | ko01230 |
| Protein processing in endoplasmic reticulum | 613 | ko04141 |
| Plant hormone signal transduction | 511 | ko04075 |
| Endocytosis | 491 | ko04144 |
| Spliceosome | 477 | ko03040 |

**Table S2 Statistical results of DEGs invovled in ABA synthesis and signal transduction**

| id | Symbol | PDT | DT | RT | DT/PDT | RT/PDT | RT/DT |
| --- | --- | --- | --- | --- | --- | --- | --- |
| Ck0038907 | NCED | 3.88 | 27.3733 | 1.38 | 2.818642569 | -1.491388385 | -4.310030955 |
| Ck0038908 | NCED | 0.8167 | 3.9867 | 0.5933 | 2.287363735 | -0.460904508 | -2.748268243 |
| Ck0053975 | NCED | 0.1833 | 10.52 | 2.19 | 5.842521776 | 3.578389847 | -2.26413193 |
| Ck0066314 | ABF2 | 7.9733 | 17.5767 | 10.2233 | 1.140406607 | 0.358610777 | -0.78179583 |
| Ck0002428 | ABF2 | 3.96 | 12.7933 | 2.6067 | 1.691819875 | -0.603294323 | -2.295114199 |
| Ck0050989 | PYL10 | 5.8133 | 1.69 | 2.2033 | -1.782342388 | -1.399677863 | 0.382664525 |
| Ck0054463 | CYP707A7 | 2.7067 | 0.0467 | 0.13 | -5.857980995 | -4.379933698 | 1.478047297 |
| Ck0031216 | PP2C06 | 12.1033 | 31.1933 | 11.3433 | 1.365833299 | -0.093559779 | -1.459393077 |
| Ck0037371 | PP2C30 | 2.8833 | 7.91 | 0.65 | 1.455940063 | -2.149226009 | -3.605166071 |
| Ck0038344 | PP2C5 | 0.58 | 1.17 | 2.8367 | 1.012383724 | 2.290071826 | 1.277688101 |
| Ck0060188 | PP2C51 | 4.74 | 19.5233 | 5.75 | 2.042240429 | 0.278674897 | -1.763565533 |
| Ck0085124 | PP2C06 | 17.96 | 38.1733 | 12.5267 | 1.08777782 | -0.519784784 | -1.607562604 |
| Ck0085126 | PP2C06 | 0.5833 | 2.0667 | 0.3567 | 1.824913293 | -0.709744125 | -2.534657419 |
| Ck0093708 | BIPP2C1 | 68.5233 | 42.9733 | 25.4067 | -0.673153646 | -1.431388226 | -0.75823458 |
| Ck0094205 | PP2C51 | 7.8267 | 23.4167 | 6.49 | 1.581065817 | -0.270179524 | -1.851245341 |
| Ck0108297 | PP2CA | 1.98 | 7.2867 | 5.2733 | 1.879758565 | 1.413214764 | -0.466543801 |
| Ck0000189 | PP2C5 | 3.6933 | 3.99 | 20.8867 | 0.111465271 | 2.499586888 | 2.388121617 |
| Ck0027154 | PYL3 | 3.0867 | 0.8567 | 0.74 | -1.849243834 | -2.060452517 | -0.211208683 |
| Ck0110597 | SNF4 | 5.5567 | 8.5567 | 3.96 | 0.622829192 | 0.00091 | 0.00570 |
| Ck0118939 | SAPK9 | 5.4667 | 11.11 | 8.4933 | 1.023125503 | 0.00423 | 0.04850 |
| Ck0056644 | SAPK2 | 26.1367 | 52.76 | 29.46 | 1.013369405 | 0.00000 | 0.08135 |
| Ck0080103 | SAPK3 | 3.38 | 10.9367 | 5.1833 | 1.694077942 | 0.00000 | 0.00064 |
| Ck0111560 | SAPK7 | 1.3433 | 0.2467 | 0.9433 | -2.445182663 | 0.00169 | 0.22608 |

**Table S3 The primes development for qRT-PCR**

| Symbol | Gene ID | Forward/Reverse (5'→3') |
| --- | --- | --- |
| GAPDH | Ck0118140 | CCCAAGATGCCTTTGAGATTACC/CTTTAACATCATTCCCAGCAGCAT |
| JMJ703 | Ck0006918 | GGAGGAGAGGAAGAAACTGAAGGG/ACCAACGCCAGAGCCAACC |
| SOC1 | Ck0045971 | ACCATGCTATCCGACCAGATTGC/TACCTCCGTTACTCCTTCAACATCG |
| ABF2 | Ck0114737 | TTAATGGTGGAGGAGGAGGAGGAG/AGCCGAAGTCTTTCCCGAATCC |
| FT1 | Ck0045127 | ACCAACAGACCGAGAAGCGAAC/GTAGACCACCGAGACCTTGCG |
| IAA8 | Ck0002629 | CGCCCCAATCAAACCCCAATTTC/CGGAATGCGGAATGCGAACTC |
| LHP1 | Ck0046481 | GGTGAGTGCTGATGCTGCTAAAAG/GGTCTCCTCATCTCTGCTCTTCTTC |
| PYRC5 | Ck0008115 | CTCGGGCAGGCTGGAGTTAC/AACCTTGTTCAATGTTCTGGGATCG |
| RHT1 | Ck0000213 | GGCAGTGAACTCGGTCTTTGAAC/TGCTCCACCACAGTCACAATCC |
